# Nitric oxide donor S‐Nitroso‐N‐acetyl penicillamine for hepatic stellate cells to restore quiescence

**DOI:** 10.1002/pdi3.95

**Published:** 2024-06-28

**Authors:** Junbao Du, Yin He, Wen Jia, Xiaohua Liang, Quan Kang, Yun He

**Affiliations:** ^1^ Stem Cell Biology and Therapy Laboratory Children's Hospital of Chongqing Medical University Chongqing China; ^2^ National Clinical Research Center for Child Health and Disorders Ministry of Education Key Laboratory of Child Development and Disorders Chongqing Key Laboratory of Pediatrics Chongqing China; ^3^ Pediatric Department University‐Town Hospital of Chongqing Medical University Chongqing China; ^4^ Department of Pediatric Surgery Children's Hospital of Chongqing Medical University Chongqing China

**Keywords:** hepatic stellate cells, lipid droplets, mitochondria, Oil Red O staining

## Abstract

Liver fibrosis is a hepatic scar repair response associated with a wide range of liver injuries, which is mediated by an imbalance between extracellular matrix (ECM) synthesis and degradation, leading to massive ECM deposition and disruption of normal liver architecture. Hepatic stellate cells (HSCs) are the main source of ECM during liver fibrosis and are the first identified cell subpopulation associated with liver fibrosis formation. Various current studies on the mechanism and treatment of liver fibrosis require resting‐state HSCs as study subjects. However, spontaneous activation of primary HSCs occurs after 2–3 days of culture after isolation, and it is common that HSCs cell lines gradually differentiate into fibroblasts with culture time. This study provides an induction medium for quiescent HSCs‐containing all‐trans retinoic acid, sodium oleate, and S‐nitroso‐N‐acetyl penicillamine (SNAP)‐and an induction method. The induction method not only maintains the HSCs cell line in a quiescent state but also restores the activated HSCs to a quiescent state. The method has a good induction effect, short induction time, and convenient operation, which is worth being popularized and used in a wide range of laboratories.

## Graphical abstract

The present study provides an induction medium for hepatic stellate cells, which can induce activated hepatic stellate cells to reverse to a quiescent state. This method is convenient and effective and has a short induction time of only 1–2 days.

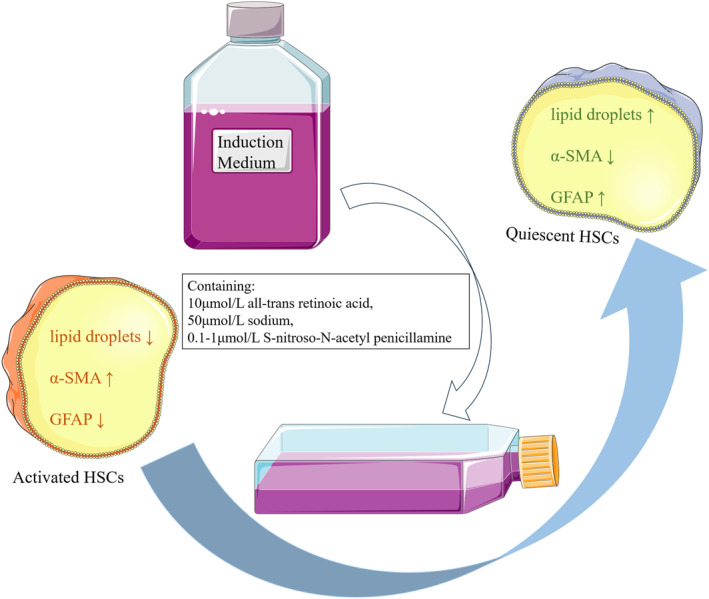

## 1 INTRODUCTION

Liver fibrosis is a hepatic scar repair response associated with chronic liver injury and can be seen in a variety of childhood disorders such as hepatitis, biliary atresia, Buerger's syndrome, nonalcoholic fatty liver disease, and hepatolenticular degeneration.^1^, ^2^, ^3^, ^4^, ^5^ The mechanism of liver fibrosis is an imbalance between extracellular matrix (ECM) synthesis and degradation, and the large amount of ECM deposited can destroy the normal liver structure.^6^, ^7^

Hepatic stellate cells (HSCs) are a type of nonparenchymal cells in the liver, accounting for 15% of the total number of intrinsic cells in the liver and about 30% of the nonparenchymal cells. HSCs are the main source of ECM in the process of hepatic fibrosis, and they are the earliest subpopulation of cells identified to be associated with the formation of hepatic fibrosis. HSCs are dispersed throughout the liver and are localized in the perisinusoidal space between hepatic sinusoidal endothelial cells and hepatocytes.^8^, ^9^ Under physiological conditions, HSCs are relatively quiescent and do not proliferate, but during liver injury, they are stimulated by a series of autocrine and paracrine fiber‐forming signals and change from a quiescent state to an activated state. The activation of HSCs is accompanied by an increase in the expression of *α*‐smooth muscle actin (α‐SMA) and the gradual disappearance of lipid droplets from the cytoplasm, a decrease in the expression of pro‐lipid genic cytokines, and an increase in the expression of receptors associated with fiber formation and cell migration, resulting in the formation of a large amount of collagen and other extracellular matrices.^10^, ^11^, ^12^ Therefore, the activation of HSCs is considered to be a central event in the process of liver fibrosis.

Consequently, targeting HSCs to inhibit their proliferative activation and induce their apoptosis has gradually become an important therapeutic strategy against liver fibrosis. This requires quiescent HSCs as the research object. However, spontaneous activation of primary HSCs occurs after 2–3 days of culture on plastic culture plates not coated with simulated biological substrates after isolation, and common cell lines HSCs gradually differentiate from lipid droplet‐rich quiescent cells to fibroblasts with the extension of the culture time, with the *α*‐SMA fraction reaching the highest peak at 15 days.^13^, ^14^, ^15^, ^16^

The present study was considered in terms of the purpose of deactivating HSCs: the process of deactivation of HSCs involves apoptosis, cellular senescence, immune clearance, and phenotype reversal. Apoptosis, cellular senescence, and immune clearance mainly reduce the number of activated HSCs, resulting in decreased cell viability. Therefore, reversal of the activated state of HSCs to the quiescent state of h HSCs by phenotypic reversal is a relatively better way.

To address the current problem of spontaneous activation of HSCs, here we provide an induction medium for quiescent HSCs‐‐containing all‐trans retinoic acid, sodium oleate, and S‐nitroso‐N‐acetyl penicillamine (SNAP)‐and an induction method. This induction method can not only maintain the cell line in the quiescent state, but also induce the activated HSCs to reverse to quiescent HSCs, which is not only applicable to LX‐2 and HSC‐T6 hepatic stellate cell lines, but also to primary HSCs cultured in vitro for more than 7 days and other hepatic stellate cell lines with the same induction effect. Moreover, this induction method, with a short induction time of only 1–2 days, is easy to operate and effective. The induced quiescent HSCs are helpful for the subsequent screening of inhibitory drugs for hepatic stellate cell activation, which is worthy of popularization and use in a wide range of laboratories.

## 2 MATERIALS & METHODS

### 2.1 Cell culture and treatment

Human Hepatic Stellate Cell Lieming Xu‐2 (LX‐2) and Rat Hepatic Stellate cell HSC‐T6 were used 16, all of which were purchased from the American Type Culture Collection (Manassas). The passaged LX‐2 or HSC‐T6 were inoculated into 24‐well plates at an initial density of 30%. After cell adhesion, LX‐2 cells, and HSC‐T6 cells were replaced with 1640 medium (Gibco) and DMEM medium (Gibco) containing 10 μmol/L all‐trans retinoic acid (Sigma Aldrich), 50 μmol/L sodium oleate (Sigma Aldrich, St. Louis, USA), 0.1–1 μmol/L S‐nitroso‐N‐acetyl penicillamine (MedChemExpress) (group b: 0.1 μmol/L SNAP; group c: 0 0.33 μmol/L SNAP; group d: 1 μmol/L SNAP), 10% fetal bovine serum (Gibco), 100 U/mL penicillin and 100 μg/mL streptomycin, respectively. The medium was changed every 24 h and incubated for 48 h.

### 2.2 Oil Red O staining and quantitative analysis

At the indicated time point, the medium was removed, and cells were washed with PBS and fixed in 4% paraformaldehyde for 20–30 min, followed by washing with PBS three times. After PBS was removed, 500 μL of 50% ethanol was added and washed for 15–20 s to remove water. Following that, Oil Red O (ORO) staining was performed according to the method of Du et al.^17^ Specifically, 200 μL of freshly prepared ORO solution (Sigma‐Aldrich, St. Louis, USA) was added to the wells and incubated for 10–15 min at room temperature. ORO solution was pipetted out, cells were washed with 500 μL of 50% ethanol for 15–20 s to remove excess dye solution, and then washed with PBS more than 3 times until the liquid was clarified, pictures were captured under a microscope within 24 h before quantitative analysis.

Furthermore, the staining effects of different solutions in cells were quantitatively analyzed according to the reported method.^18^ Briefly, after complete washing and drying, 100 μL of 100% ethanol was added to each well and incubated on a shaker for 10 min at room temperature to release the ORO from the stained cells. Finally, 50 μL of the ORO‐containing extract was transferred to a 96‐well plate, and the absorbance at a wavelength of 492 nm was measured with a microplate reader. All the experiments were independently repeated at least three times.

### 2.3 Immunofluorescence assay

HSCs were seeded in a 24‐well plate with 48 h of different treatments, then fixed in 4% paraformaldehyde for 30 min, followed by permeabilized with 0.3% Triton X‐100 (Solarbio) for 15 min and blocked with 5% albumin from bovine serum (Solarbio) for 20 min at room temperature, followed by incubating primary antibodies of GFAP (ET1601‐23, HuaBio) and *α*‐SMA (HA600032, HuaBio). After gently washing with PBS, cells were subsequently incubated with appropriate secondary fluorescent antibodies (ZSGB‐BIO, Beijing, China) for 1 h at room temperature. The nucleus was stained with DAPI (Solarbio, Beijing, China). The presence of the proteins was ascertained under the laser confocal microscope (Nikon, Tokyo, Japan) and semi‐quantitative analyses were then performed following the method of Jensen et al.^19^

### 2.4 Immunoblot analysis

For the collection of protein, cells were washed with cold PBS and lysed by the addition of RIPA buffer for 20 min at 4°C. Insoluble materials were removed by centrifugation at 1500 G for 15 min at 4°C. The supernatant was saved and the protein concentration was determined using a Bio‐Rad protein assay kit (Bio‐Rad, Hercules, CA). An identical amount of protein (30 μg) from each lysate was subjected to 12.5% SDS–polyacrylamide gel electrophoresis (PAGE). The fractionated proteins were transferred to polyvinylidene fluoride membranes (Millipore), and the filters were blocked for 1.5 h using non‐fat dried milk in Tris‐buffered saline (TBS: 50 mM Tris, 0.15 M NaCl, pH 7.5) containing 0.1% Tween 20, washed with TBS, and then incubated overnight at 4°C with a primary antibody at a 1:500 dilution of mouse anti‐α‐SMA (HA600032, HuaBio), GAPDH (M1310‐2, HuaBio) and rabbit anti‐GFAP (ET1601‐23, HuaBio) monoclonal antibodies (mAb). The membranes were further incubated with the appropriate second antibody (ZSGB‐BIO) at room temperature for 2 h and visualized using enhanced chemiluminescent substrate (Bio‐Rad) under the ChemiDoc Touch Imaging System (Bio‐Rad). Images from the densitometer were transferred to a personal computer and analyzed using Science Lab software (Fuji Film). The density of the target protein band of untreated cells was assigned the value of 1.0 and each result was calculated as relative units.

### 2.5 Transmission electron microscopy

Cells were washed with phosphate‐buffered saline solution (PBS) twice and then digested with 0.25% trypsin‐EDTA solution (C0201, Beyotime). Cells were loaded into 1.5‐mL centrifuge tubes and centrifuged at 1200 rpm for 10 min; 4%154Journal Pre‐proof glutaraldehyde solution was added along the walls of the tubes. The cell samples were subsequently rinsed with distilled water, dehydrated with a gradient of ethanol and methanol, and then soaked and embedded in epoxy resin. Finally, the ultrathin sections were randomly observed using a transmission electron microscope (H‐7500).

### 2.6 Statistical analysis

All data were expressed as mean ± standard deviation (SD) and were analyzed using GraphPad Prism 9.0 software (GraphPad Software, Inc.). Statistical analyses were performed using a two‐tailed Student's *t*‐test to determine the significance of differences between the two groups. The one‐way ANOVA was used to measure the significance of differences among more than two groups. A *p*‐value <0.05 was considered statistically significant.

## 3 RESULTS

### 3.1 Screening for optimal SNAP induction concentration

**FIGURE 1:**
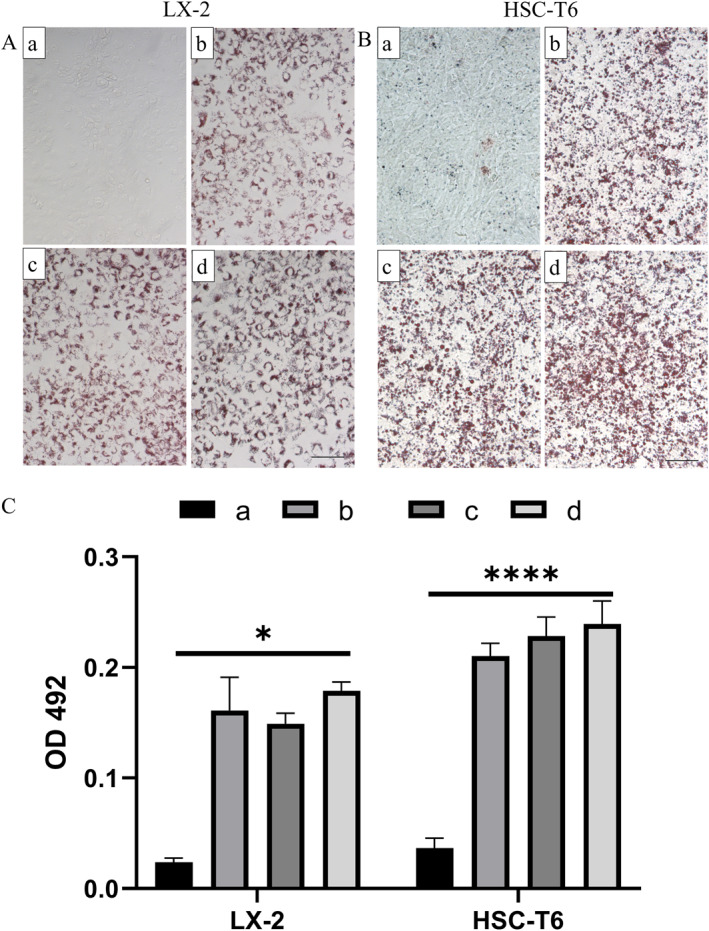
ORO staining after induction by different induction methods (A and B) Representative images under the light microscope of ORO staining for LX‐2 (A) and HSC‐T6 (B) cells induced by different induction methods. Scale bars: 100 μm. (C) The absorbance at 492 nm of ORO‐containing extracts semi‐quantitatively reflects the staining effect of different groups. All the experiments were independently repeated three times. (*n* = 3. Error bars indicate mean ± SD. *p < 0.05; by ANOVA for multiple comparisons).

In various mouse models of liver disease and in vitro cultured HSCs, it was observed that during activation of HSCs, triglycerides are hydrolyzed to free fatty acids (FIFA), which undergo *β*‐oxidation to generate succinate, and then ATP is generated through the tricarboxylic acid cycle (TCA) to provide energy, resulting in a marked decrease in the gradual disappearance of intracytoplasmic lipid droplets and a concomitant decrease in the expression of pro‐lipogenic factors. Lipid droplets are easily stained by ORO staining (which is the gold standard for lipid droplet staining), so HSCs in the resting and activated states can be distinguished from those in the activated state by morphological features.^20^, ^21^ Therefore, in this study, the content of lipid droplets in HSCs was examined as one of the indicators to determine their activation. The results are shown in Figure 1A,B, with little ORO staining and small lipid droplets in the control group (a) in HSC‐T6 cells and almost no ORO staining in the control group (a) in LX 2 cells. The volume of lipid droplets in the HSCs of induction groups (b), (c), and (d) increased significantly, and the oil‐red staining increased; group (d) was the most effective, and group (d) induction protocol was chosen for subsequent experiments.

Further, 100 μL of 100% ethanol was added to each well to elute intracellular ORO dye, and 50 μL of the eluate was taken into a 96‐well plate, and the OD value at 492 nm was detected by an enzyme marker. The results were shown in Figure 1C, the OD492 value of ORO eluate in the induced group was significantly higher than that of the control group (*p* < 0.05). The semi‐quantitative results were consistent with the results of cell staining observed under light microscopy.

### 3.2 Detection of activation‐related markers in HSCs after induction

**FIGURE 2:**
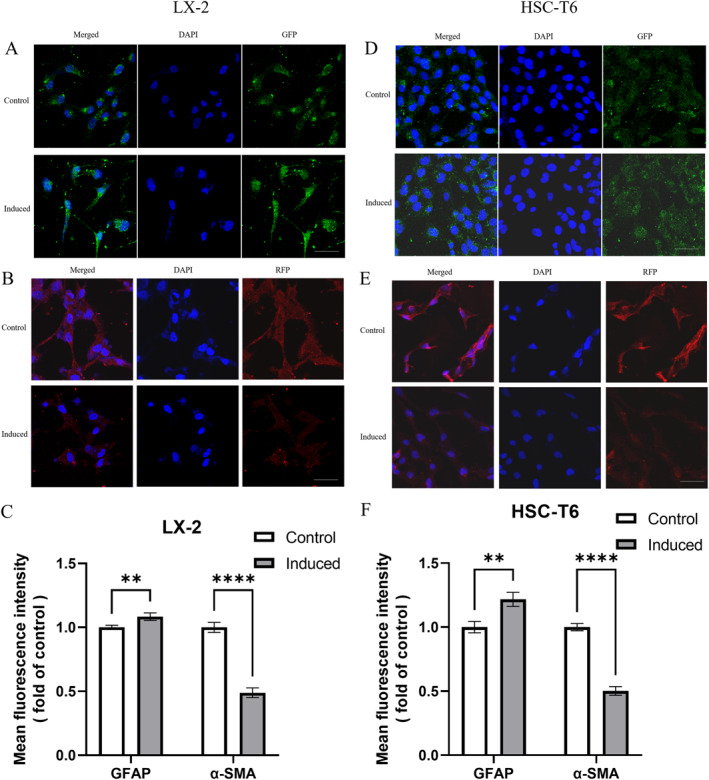
Changes in protein expression in HSC‐T6 and LX‐2 cells before and after induction detected by immunofluorescence assay. (A and D) Representative immunofluorescence micrographs of LX‐2 (A) and HSC‐T6 (D) cells stained for GFAP before and after induction. Scale bars, 50 μm. (B and E) Representative immunofluorescence micrographs of LX‐2 (B) and HSC‐T6 (E) cells stained for *α*‐SMA before and after induction. Scale bars, 50 μm. (C and F) Quantification of GFAP and *α*‐SMA protein from LX‐2 (C) and HSC‐T6 (F) cells before and after induction by mean fluorescence intensity. Representative of 3 photographs for every group and the mean was estimated. (*n* = 3. Error bars indicate mean ± SD. **p < 0.01; ****p < 0.001, by Student's *t*‐test between 2 groups comparisons).

Immunofluorescence was used to detect *α* smooth muscle actin (α‐SMA) and glial fibrillary acidic protein (GFAP) expression in HSCs before and after induction. The results are shown in Figure 2, where red fluorescence indicates the expression of *α*‐SMA and green fluorescence indicates the expression of GFAP. In the control group, both LX 2 (Figure 2A–C) and HSC‐T6 (Figure 2D–F) cells highly expressed *α*‐SMA, and the red fluorescence was more pronounced in LX 2, with a lower level of GFAP expression. On the contrary, the red fluorescence expression of *α*‐SMA was significantly reduced and the green fluorescence expression of GFAP was significantly enhanced in both HSCs after 48 h of induction.

**FIGURE 3:**
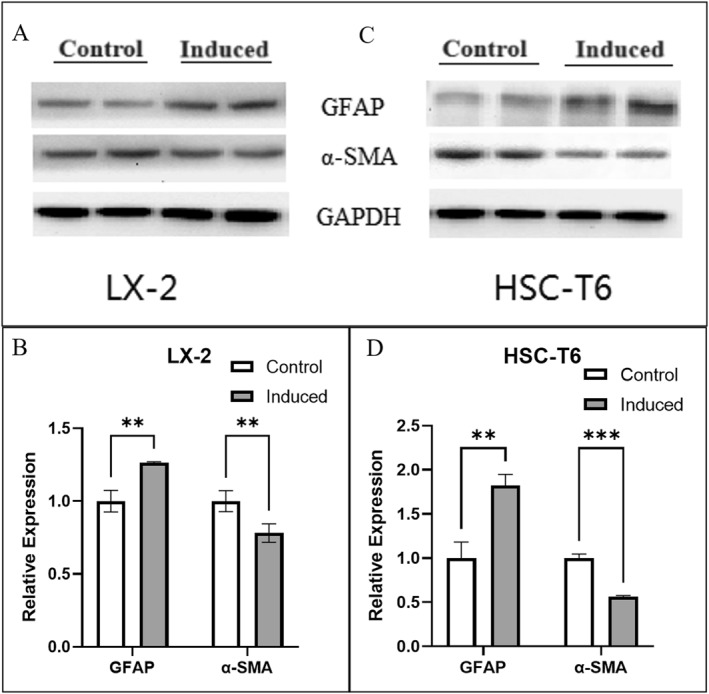
Changes in protein expression in HSC‐T6 and LX‐2 cells before and after induction detected by western blot. (A and C) Representative western blot images of protein expression from LX‐2 (A) and HSC‐T6 (C) cells before and after induction. (B and D) Quantification of GFAP and *α*‐SMA protein from LX‐2 (B) and HSC‐T6 (D) cells before and after induction. All the experiments were independently repeated at least three times. (*n* = 3. Error bars indicate mean ± SD. **p < 0.01; ***p < 0.001, by Student's *t*‐test between 2 groups comparisons).

Western blot was used to detect the expression of HSCs marker proteins, and ImageJ software was used to analyze the grayscale values of the Western blot bands after the bands were obtained using ECL exposure. The results, as shown in Figure 3, showed that either LX 2 or HSC‐T6 cells highly expressed *α*‐SAM, while the basal expression level of GFAP was low. On the contrary, *α*‐SMA expression was significantly decreased and GFAP expression level was significantly increased in both HSCs after 48 h of induction (*p* < 0.05). The above results indicate that this induction method reduced the expression of *α*‐SMA, an activation marker of HSCs, and restored the expression of GFAP, a marker of the quiescent phase of HSCs, which can induce the activated HSCs to revert to the quiescent phase.

### 3.3 Recovery of subcellular structures of HSCs after induction

**FIGURE 4:**
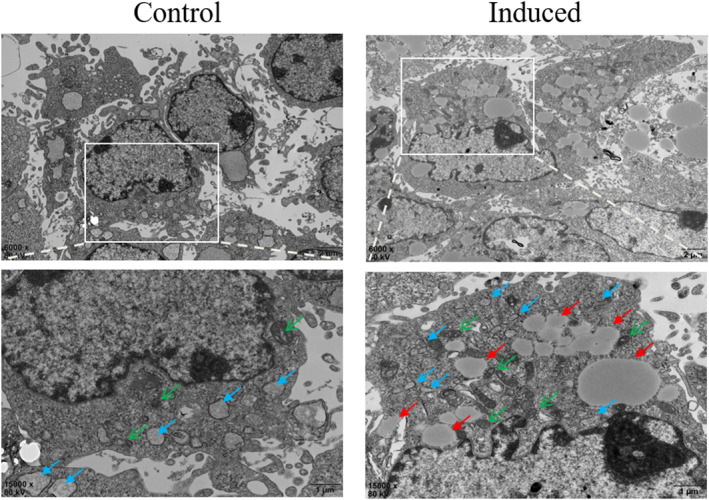
Representative transmission electron micrographs of subcellular structures of HSC‐T6 cells before and after induction (blue arrows: endoplasmic reticulum; red arrows: lipid droplets; green arrows: mitochondria). Scale bars, 2 μm (top) and 1 μm (bottom).

The induced HSC‐T6 cells were taken, fixed sectioned, and then photographed using transmission electron microscopy to observe the changes in subcellular structure. As shown in Figure 4 (blue arrows: endoplasmic reticulum; red arrows: lipid droplets; green arrows: mitochondria), the endoplasmic reticulum pool of the control group was enlarged, dark‐colored, and contained many proteins with vigorous protein synthesis, and the mitochondrial ridges were visible with a normal structure, with occasional lipid droplets. After induction, the endoplasmic reticulum expansion of the cells was significantly reduced, mitochondria were solidified, the ridge gap was enlarged, the density of dorsal protrusions was increased, the color was dark, and the intracellular lipid droplets were increased and of different sizes, and glycogen was increased. It is suggested that the protein synthesis activity of the induced HSCs was reduced, the lipid droplet storage was increased, and the restoration to the quiescent phase was achieved.

## 4 DISCUSSION

Liver fibrosis is a repair response of the liver to acute and chronic liver injury caused by inflammation, viruses, and other factors, and can further develop into cirrhosis or hepatocellular carcinoma, which can be seen in a variety of children's diseases such as hepatitis, biliary atresia, Bulgular syndrome, non‐alcoholic fatty liver, and hepatolenticular degeneration, which is a serious threat to children's life and health. Hepatic fibrosis undergoes (1) liver injury caused by various factors; (2) release of factors (activators); (3) activation and proliferation of hepatic stellate cells (HSCs); (4) collagen synthesis, secretion, and modification; and (5) imbalance in the accumulation and removal of extracellular collagen fibers. It is mainly characterized by excessive deposition of extracellular matrix (ECM) in the liver, leading to structural changes and loss of liver function.^22^, ^23^

HSCs account for 15% of the total number of intrinsic hepatic cells and about 30% of the nonparenchymal cells, and are present in the lumen of the Disse, in the shape of a stellate or polygonal, with multiple vitamin A‐rich lipid droplets in the cytoplasm (so they are also known as lipid‐storing cells), and with their elongated protrusions extending outward around the outside of the blood sinusoidal endothelial cells, which are the primary site of storage of retinoid derivatives in vivo. In normal liver, stellate cells are resting, do not express *α*‐smooth muscle actin (α‐SMA, encoded by the *ATCA2* gene), have low proliferative activity and a low capacity to synthesize collagen, and their main function is to store retinoids.^8^, ^9^

However, when the liver is stimulated by pathological factors such as physical, chemical, and microbial infections, HSCs proliferate and activate into their activated form, myofibroblasts (MFBs). The release of lipid droplets (release of vitamin A) after activation of HSCs is a natural defense mechanism to maintain homeostasis in the liver. Vitamin A uptake by hepatocytes and somatic and adipocytes helps FXRs and PPARs to fulfill their biological functions, for example, to reduce intracellular lipid accumulation in hepatocytes, accelerate lipid metabolism, and redistribute lipids to organs other than the liver 10–12. Afterward, activated HSCs begin to synthesize and secrete large amounts of cytokines, chemokines, and growth factors in large quantities, showing marked cell proliferation, increased migratory adhesion and contractility, large expression of α‐SMA, and the production of large quantities of ECM, which promotes the onset and progression of hepatic fibrosis. HSCs are the main source of the extracellular matrix, and therefore the activation of HSCs is considered the central event in hepatic fibrosis development.

Accordingly, HSCs as a target to inhibit their proliferation and activation and induce their apoptosis have gradually become an important therapeutic strategy against liver fibrosis. This requires the non‐activated state of HSCs as a research object. However, spontaneous activation of primary HSCs occurred after 2–3 days of culture on plastic culture plates not coated with a simulated biological substrate after isolation, and the hepatic stellate cell lines LX‐2 and HSC‐T6 gradually differentiated from lipid droplet‐rich quiescent cells to fibroblasts with the prolongation of culture time, and *α*‐SMA secretion reached the highest peak at 15 days 13–16.

Some studies have reported that HSCs can be maintained in a quiescent state by culturing primary HSCs onto basement membrane matrix‐coated culture plates and that drugs promoting adipocyte differentiation can change activated HSCs into a quiescent state. In addition, the liver is an important site for glucose metabolism. It has been found that decreased hepatic glycogen synthesis in the liver is accompanied by a significant increase in all indicators of liver fibrosis, and rosiglitazone and insulin promote hepatic glycogen synthesis and inhibits hepatic glucose isomerization. Some studies have used the combination of sodium oleate, all‐trans retinoic acid, and rosiglitazone or the combination of sodium oleate, all‐trans retinoic acid, and insulin to restore HSCs to a resting state.^24^, ^25^, ^26^, ^27^ However, the effect of restitution was poor, with too high a concentration of sodium oleate and too large a lipid droplet leading to cell rupture.

Nitric oxide (NO) is a biological medium commonly found in various cells of vertebrates and is an important regulator of intercellular information transfer.^28^ Nitric oxide synthases (NOs) are widely distributed in hepatocytes, blast cells, and hepatic sinusoidal endothelial cells, and can be activated by lipopolysaccharide (LPS), endotoxin, and a variety of cytokines, generating a large amount of endogenous NO with cytotoxicity, which is involved in the pathological process of various liver diseases.^29^, ^30^ However, some studies have reported that NO has a protective effect in the process of liver fibrosis. High concentrations of NO inhibit the activation of HSCs, resulting in a reduction of collagen synthesis and alleviation of extracellular matrix deposition to alleviate hepatic fibrosis.^31^, ^32^, ^33^, ^34^ Therefore, NO has a dual biological activity of cytotoxic effect and cytoprotective effect depending on the concentration of NO. S‐nitroso‐N‐acetyl penicillamine (SNAP) is a nitrosothiol derivative that acts as an NO donor and releases nitric oxide. SNAP is a potent vasodilator in vitro, inhibiting mitosis and spreading of vascular smooth muscle cells, and also acts as platelet aggregation, also acts as a stabilizing inhibitor of platelet aggregation. In addition to their vasodilatory properties, NO donors may exert direct antifibrotic properties and have been used clinically in the treatment of hepatomegaly.^35^, ^36^, ^37^, ^38^

To solve the current problem of spontaneous activation of HSCs, we treated the cells by adding different concentrations of SNAP to the traditional recovery method. Then it was compared with the cells without any treatment and the cells treated by the traditional method. The recovery effect was evaluated by detecting the content of intracellular lipid droplets, hepatic stellate cell protein markers, and subcellular structure by electron microscopy after treatment. We found that: the induction medium acted against activated‐phase HSCs for 48 h, significantly increased lipid droplet storage in HSCs, decreased the expression of the activation marker *α*‐SMA, and restored the expression of the quiescent‐phase marker GFAP, which could induce activated HSCs to revert to the quiescent phase. In terms of subcellular structure, after induction, the expansion of the endoplasmic reticulum was significantly reduced, mitochondria were fixed and contracted, the ridge gap was enlarged, the density of dorsal protrusions was increased and dark in color, and the intracellular lipid droplets were increased and varied in size, and glycogen was increased. It is suggested that the protein synthesis activity of HSCs decreased and lipid droplet storage increased after induction.

Although a good response was achieved, there are still some imperfections in our study. Currently, the activation and proliferation of HSCs cause liver fibrosis as well, and the reversal mechanism of liver fibrosis is still unclear. Further research on the mechanism of cytokines, chemokines, transcription factors, etc. on the regulation of HSCs is needed to find the signal transduction pathways during the activation of HSCs, to develop drugs to inhibit the activation of HSCs or promote their apoptosis.

## 5 CONCLUSIONS

In summary, this induction method can be used not only to maintain cell lines in a quiescent state but also to induce the reversal of activated HSCs to a quiescent state. It is not only suitable for LX‐2 and HSC‐T6 hepatic stellate cell lines, but also for primary HSCs and other HSCs cultured for more than 7 days in vitro. Moreover, this induction method, with a short induction time of only 1–2 days, is easy to operate and effective. The induced quiescent HSCs are helpful for the subsequent screening of inhibitory drugs for the activation of HSCs, which is of great significance for the research on the mechanism of liver fibrosis occurrence, diagnosis, and treatment. It is worthwhile to promote its use in a wide range of laboratories.

## AUTHOR CONTRIBUTIONS

Junbao Du performed the experiments, completed the overall data analysis, and wrote the manuscript; Yin He performed the experiments and completed the overall data analysis; Wen Jia read the manuscript; Kang Quan and Yun He were responsible for the overall idea of the project and guiding the direction of the research, as well as revising the manuscript.

## CONFLICT OF INTEREST STATEMENT

No potential conflict of interest was reported by the author(s).

## ETHICS STATEMENT

Not applicable.

## CONSENT FOR PUBLICATION

Not applicable.

## Data Availability

The data that support the findings of this study are available from the corresponding author upon reasonable request.
